# Early differentiation of long-standing persistent atrial fibrillation using the characteristics of fibrillatory waves in surface ECG multi-leads

**DOI:** 10.1038/s41598-019-38928-6

**Published:** 2019-02-26

**Authors:** Junbeom Park, Chungkeun Lee, Eran Leshem, Ira Blau, Sungsoo Kim, Jung Myung Lee, Jung-A Hwang, Byung-il Choi, Moon-Hyoung Lee, Hye Jin Hwang

**Affiliations:** 1 Department of Cardiology, College of Medicine, Ewha Womans University, Seoul, South Korea; 2 Cardiovascular Devices Division, National Institute of Food and drug safety Evaluation, Cheongju-si, South Korea; 3 Cardiovascular Division, Department of Medicine, Beth Israel Deaconess Medical Center, Boston, MA United States; 4 Department of Cardiology, Medical College of Wisconsin, Milwaukee, WI USA; 5 Department of Internal Medicine, College of Medicine, Yonsei University, Seoul, South Korea; 6 Division of Cardiology, Kyung Hee University Medical College, Seoul, South Korea; 7 Division of Cardiology, Yonsei University Health System, Seoul, South Korea

## Abstract

We characterized the f-waves in atrial fibrillation (AF) in the surface ECG by quantifying the amplitude, irregularity, and dominant rate of the f-waves in leads II, aVL, and V_1_, and investigated whether those parameters of the f-waves could discriminate long-standing persistent AF (LPeAF) from non-LPeAF. A total of 224 AF patients were enrolled: 112 with PAF (87 males), 48 with PeAF (38 males), and 64 with LPeAF (47 males). The f-waves in surface ECG leads V_1_, aVL, and II, which reflect well electrical activity in the right atrium (RA), the left atrium (LA), and both atria, respectively, were analyzed. The f-waves for LPeAF had lower amplitudes in II and aVL, increased irregularity and a higher dominant rate in II and V_1_ compared to PAF and PeAF (all p < 0.02). In a multivariate analysis, a low amplitude in lead II (<34.6 uV) and high dominant rate in lead V_1_ (≧390/min) (p < 0.00_1_) independently discriminated LPeAF from the other AF types. The f-waves combined with both a low amplitude in lead II and high dominant rate in lead V_1_ were significantly associated with LPeAF (OR 6.27, p < 0.001). Characteristics of the f-waves on the surface ECG could discriminate LPeAF from other types of AF.

## Introduction

The activity of atrial fibrillary waves (f-waves) is thought to be chaotic and disorganized. This irregular spatiotemporal pattern could be influenced by various underlying mechanisms including anatomical and pathological changes in the atrial substrate, electrophysiological dynamics such as conduction delays, refractoriness dispersion, and an imbalanced autonomic tone^1–3^. Atrial fibrillation (AF) is regarded to be initiated by premature atrial beats and maintained by continuous wave breaks and subsequent wave formation due to a combination of dynamic instability, anatomical fixed obstacles, triggered activity^4^, and high frequency sources^5^ from specific anatomical sites^6^. These dynamic activities interacting with anatomical or pathological substrates are manifested as f-wave patterns on the surface electrocardiogram (ECG)^7–9^. On the other hand, the clinical AF types (paroxysmal, persistent, and long-standing persistent), which are determined by the duration and treatment response, have been shown to be related to the left atrial size and stiffening of the atria^10,11^, suggesting that fibrillation sustainability could be largely influenced by atrial structural- pathological changes. Indeed, atrial samples obtained from patients with long-standing persistent AF (LPeAF) have demonstrated abundant fibrosis, inflammatory infiltrates, and disorganized myofiber arrays, which may directly result in more conduction delays or wave breaks during fibrillation^12^. The interaction between the electrical activity and fixed substrates could affect the rate, size, or irregularity patterns of f-waves, presumably allowing for discrimination of the different clinical AF types.

A relatively low f-wave amplitude could represent a small amount of activated muscle fibers since the voltage is theoretically proportional to the current amount generated from the conductive myocardial mass, which would occur more frequently in a chamber with scant myocardial bundles. Fine, fast oscillating f-waves might reflect a rapid turn-over of the generation and annihilation of small wavelets, which are dynamically formed by substrates that can make more wavelets by breaking down preceding wavelets in either dynamic fashion or with anatomical obstacles. Enlarged atria, highly susceptible to LPeAF, could give rise to very slow conduction and thereby small wavelets with a small wavelength^13^. It implies that the features of the f-waves might differ in paroxysmal (PAF), persistent (PeAF), and LPeAF. According to the 2017 expert consensus statement on catheter and surgical ablation of AF^14^, differentiating LPeAF from other AF types is clinically important in predicting the outcome, since LPeAF is usually refractory or has a poor outcome with treatments for maintaining sinus rhythm, such as catheter ablation or surgical ablation, in contrast to PAF or PeAF. The current clinical practice guidelines (European Society of Cardiology, American Heart Association guideline)^15,16^ recommend catheter ablation as a curable or substrate-modifying treatment for AF. Therefore, the analysis of the f-wave characteristics by a simple and non-invasive method such as a surface ECG could assist physicians in making a diagnosis and setting a treatment plan. We attempted to characterize the f-waves in the surface ECG by quantifying the amplitude, irregularity, and dominant rate of the f-waves in leads II, aVL, and V_1_, and investigated whether those parameters of the f-waves could discriminate LPeAF.

## Results

### Baseline characteristics

A total of 224 patients (76.8% male, 61.4 ± 12.3 years) were included in this study. The number of patients with PAF, PeAF, and LPeAF was 112 (50%), 48 (21.4%), and 64 (28.6%), respectively. The baseline characteristics are described in Table 1. Patients with LPeAF were older (65.3 ± 9.3 years, p < 0.001), had a longer AF duration (7.5 ± 2.5 years, p = 0.007) than the non- LPeAF patients, including PAF and PeAF patients (Table 1). However, no differences in the gender, body mass index (BMI), or underlying disease among the groups were observed. There were no statistically significant differences in the heart rate, QRS duration, QT, and QTc interval in the 12-lead surface ECG (Table 1) among the different AF types.

**Table 1:** Baseline characteristics.

	All patients	PAF	PeAF	LPeAF	P value
**Number of Subjects (n)**	224	112	48	64	
Age (years)	61.4 ± 12.3	61.6 ± 12.5	55.6 ± 13.1	65.3 ± 9.3	<0.001
Male gender, n (%)	172 (76.8)	87 (77.7)	38 (79.2)	47 (73.4)	0.742
AF duration (years)	6.7 ± 2.5	6.3 ± 2.4	6.5 ± 2.7	7.5 ± 2.5	0.007
Body surface area (m^2^)	1.8 ± 0.2	1.8 ± 0.2	1.8 ± 0.2	1.7 ± 0.1	0.025
Body mass index (kg/m^2^)	24.2 ± 2.8	24.2 ± 2.9	24.7 ± 2.5	24.0 ± 2.7	0.492
**Comorbidities**					
Chronic Heart Failure, n (%)	10 (41.5)	6 (5.4)	2 (4.2)	2 (3.1)	0.783
Hypertension, n (%)	93 (41.5)	46 (41.1)	18 (37.5)	29 (45.3)	0.702
Diabetes, n (%)	28 (12.5)	12 (10.7)	5 (10.4)	11 (17.2)	0.406
Cerebrovascular accident (incl. TIA), n (%)	19 (8.0)	9 (8.0)	1 (2.1)	9 (14.1)	0.077
Coronary disease, n (%)	18 (8.0)	9 (8.0)	4 (8.3)	5 (7.8)	0.995
Dyslipidemia, n (%)	35 (15.6)	21 (18.8)	5 (10.4)	9 (14.1)	0.38
Valvular heart disease, n (%)	6 (2.7)	2 (1.8)	2 (4.2)	2 (3.1)	0.671
**Electrocardiography (ECG)**					
Heart rate (/min)	71.5 ± 26.5	71.8 ± 23.3	69.3 ± 24.1	72.8 ± 34.1	0.801
QRS duration (ms)	102.3 ± 58.3	98.9 ± 16.2	119.8 ± 121.2	93.7 ± 12.0	0.068
QT (ms)	411.1 ± 245.0	430.5 ± 326.1	401.0 ± 46.3	374.4 ± 51.5	0.402
QTc (ms)	427.2 ± 23.0	428.4 ± 31.5	431.2 ± 29.8	420.5 ± 25.9	0.193

PAF; paroxysmal atrial fibrillation, PeAF; persistent atrial fibrillation, LPeAF; long-standing persistent atrial fibrillation, TIA; transient ischemic attack.

### Overall characteristics of the f-waves in leads II, aVL, and V_1_

Anatomically, lead II aligned with the interatrial septum, and aVL aligned with the lateral side of the LA, and V_1_ was close to the RA (Sup. Fig. 1). We found that the amplitude, irregularity index, and dominant rate of the f-waves differed significantly among these leads (Supplementary Table 1). Compared to lead II and aVL, the f-waves in the lead V_1_ presented the highest amplitude (37.5 ± 17.5 uV in II, 23.1 ± 8.4 uV in aVL, and 47.5 ± 33.4 uV in V_1_) and a high dominant rate (316.1 ± 124.4/min in II, 340.3 ± 107.8/min in aVL, and 353.1 ± 117.7/min in V_1_). The irregularity of the f-waves (measured as ApEn) was prominent in lead aVL as compared to the other two leads, leads II and V_1_ (0.12 ± 0.02 in II, 0.13 ± 0.02 in aVL, and 0.12 ± 0.02 in V_1_).

### The amplitude of f-waves

Overall, the f-wave amplitude was significantly lower in LPeAF than non- LPeAF and was similar between PAF and PeAF in all leads examined (Table 2). The differences in the f-wave amplitude were prominent in lead II (PAF vs. PeAF vs. LPeAF; 41.3 ± 17.2 uV vs. 41.2 ± 21.4 uV vs. 28.0 ± 9.8 uV, p < 0.001) and lead aVL (Table 2). However, the f-waves amplitude in lead V_1_ was not statistically significant (Table 2).

**Table 2:** Difference in the fibrillary waves according to the AF type.

	PAF	PeAF	LPeAF	p	(1)	(2)	(3)	
Number of patients	107	42	59	208				
Lead II	Amplitude (uV)	41.26 ± 17.23	41.20 ± 21.38	28.03 ± 9.76	<0.001	1	<0.001	<0.001
	Irregularity	0.12 ± 0.02	0.12 ± 0.02	0.13 ± 0.02	0.012	0.355	0.915	0.008
	Dominant rate (DF, rate/min)	286.33 ± 121.38	364.29 ± 110.76	335.42 ± 126.07	0.001	0.001	0.713	0.039
Lead aVL	Amplitude (uV)	24.43 ± 7.87	24.03 ± 11.15	19.72 ± 5.80	0.01	0.667	0.04	0.003
	Irregularity	0.13 ± 0.02	0.14 ± 0.03	0.13 ± 0.02	0.271	0.141	1	0.442
	Dominant rate (DF, rate/min)	325.85 ± 102.59	356.69 ± 109.80	356.07 ± 114.23	0.108	0.379	1	0.304
Lead V_1_	Amplitude (uV)	49.41 ± 39.70	52.48 ± 31.70	40.55 ± 15.35	0.177	1	0.299	0.338
	Irregularity	0.12 ± 0.02	0.12 ± 0.03	0.13 ± 0.02	0.013	1	0.014	0.003
	Dominant rate (DF, rate/min)	330.07 ± 119.37	343.20 ± 136.38	404.32 ± 82.39	0.001	1	0.043	<0.001

p-value of (1): PAF vs. PeAF, (2): PeAF vs. LPeAF, (3) LPeAF vs. PAF.

PAF; paroxysmal atrial fibrillation, PeAF; persistent atrial fibrillation, LPeAF; long-standing persistent, TIA; transient ischemic attack.

### The irregularity of f-waves

The severity of the irregularity was prominent in leads II and V_1_, where the f-waves of LPeAF were more irregular than those of PAF and PeAF (PAF vs. PeAF vs. LPeAF: 0.12 ± 0.02 vs. 0.12 ± 0.02 vs. 0.13 ± 0.02, p = 0.012 in lead II; 0.12 ± 0.02 vs. 0.12 ± 0.03 vs. 0.13 ± 0.02, p = 0.013 in lead V_1_). However, there was no significant difference in lead aVL (Table 2).

### The dominant rate of f-waves

In lead II, LPeAF had a higher dominant rate compared to PAF (286.3 ± 121.4/min in PAF vs. 335.4 ± 126.1/min in LPeAF, p = 0.039). The f-waves in lead II in PeAF had a similar dominant rate as compared to LPeAF (p > 0.05) and faster dominant rate than in PAF (p = 0.001). This was the only remarkable difference in the f-wave characteristics between PAF and PeAF. An analysis of lead V_1_ of the dominant rate showed a faster dominant rate in LPeAF than non- LPeAF (PAF vs. PeAF vs. LPeAF: 330.1 ± 119.4/min vs. 343.2 ± 136.4/min vs. 404.3 ± 82.4/min, respectively; p < 0.001), while the dominant rate between PAF and PeAF was similar (p > 0.05) (Table 2). The dominant rate in lead V_1_ revealed remarkable differences in discriminating LPeAF.

### The multivariate analysis

Table 3 shows the multivariate analysis for differentiating LPeAF according to the amplitude, irregularity, and dominant rate in leads II, aVL and V_1_. In model I, including f-wave variables only in lead II, the amplitude index was the most powerful predictor of LPeAF (RMS, OR = 0.909, 95% CI 0.871–0.949, p < 0.001). In model II with aVL, the amplitude index was also the strongest predictor of LPeAF (RMS; OR = 0.908, 95% CI 0.856–0.964, p = 0.001), and in model III with lead V_1_, the dominant rate was the strongest predictor of LPeAF (OR = 1.382, 95% CI 1.057–1.807, p = 0.018).

**Table 3:** Uni- and multivariate analyses for predicting LPeAF.

		Univariate analysis			Multivariate analysis (Model I)			Multivariate analysis (Model II)			Multivariate analysis (Model III)			Multivariate analysis (Model IV)		
		OR	95% CI	*p*	OR	95% CI	*p*	OR	95% CI	*p*	OR	95% CI	*p*	OR	95% CI	*p*
	**Age**	1.04	1.013–1.068	0.003	1.032	0.999–1.066	0.054	1.032	0.999–1.067	0.06	1.036	1.003–1.070	0.033	1.033	0.995–1.072	0.088
	**Male**	0.774	0.396–1.512	0.454	1.185	0.534–2.631	0.676	0.752	0.333–1.697	0.492	0.936	0.414–2.114	0.936	0.952	0.392–2.314	0.914
	**AF duration**	1.194	1.065–1.340	0.002	1.213	1.059–1.388	0.005	1.234	1.075–1.415	0.003	1.176	1.028–1.345	0.018	**1.265**	**1.087–1.471**	**0.002**
	**BSA (m** ^**2**^ **)**	0.198	0.029–1.368	0.101												
	**BMI (kg/m** ^**2**^ **)**	0.956	0.846–1.081	0.475												
**Lead II**	**Amplitude**	0.909	0.875–0.943	<0.001	0.909	0.871–0.949	<0.001							**0.905**	**0.863–0.950**	**<0.001**
	**Irregularity**	1	1.000–1.000	<0.001	38.465	0.001~	0.763									
	**Dominate rate**	1.115	0.959–1.296	0.157	1.018	0.837-0.238	0.862									
**Lead aVL**	**Amplitude**	0.91	0.861–0.962	0.001				0.908	0.856–0.964	0.001						
	**Irregularity**	1	1.000–1.000	0.004				0.003	0.001~	0.512						
	**Dominate rate**	1.121	0.933–1.348	0.223				1.124	0.906–1.393	0.288						
**Lead V**_**1**_	**Amplitude**	0.977	00957–0.998	0.03							0.984	0.962–1.007	0.182			
	**Irregularity**	1	1.000–1.000	0.108							538678.93	0.001~	0.229			
	**Dominate rate**	1.457	1.186–1.789	<0.001							1.382	1.057–1.807	0.018	**1.405**	**1.115–1.770**	**0.004**

Amplitude, irregularity, and dominant rate were calculated by root mean square (RMS), approximate entropy, and dominant rate (DF), respectively.

The multivariate analysis in model IV was adjusted for the age, gender, AF duration, amplitude in lead II, and dominant rate (DF) in lead V_1_.

BSA; body surface area, BMI; body mass index.

### The combination of the amplitude in lead II and dominant rate in lead V_1_ had a high predictive value of LPeAF

**Figure 1:**
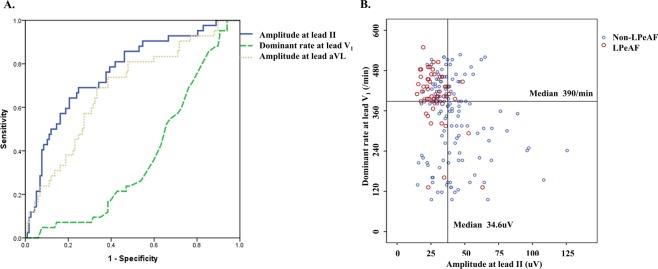
The prediction of LPeAF. (**A**) Area under the curve of the f-waves for predicting LPeAF. (**B**) The sensitivity and specificity of a low amplitude (RMS) in lead II and high dominant rate (DF) in lead V_1_. LPeAF; long-standing persistent atrial fibrillation, Dominant rate; DF, root mean square; RMS.

The age, gender, AF duration, and only three of the strongest f-waves parameters, including the amplitude indices in leads II and aVL and dominant rate in V_1_, were included in model IV. Finally, the amplitude index in lead II (RMS: OR = 0.905, 95% CI 0.863–0.950, p < 0.001) and dominant rate in lead V_1_ (DF: OR = 1.405, 95% CI 1.115–1.770, p = 0.004) were the most powerful parameters for differentiating LPeAF from non- LPeAF. The area under the curve (AUC) for predicting LPeAF was 0.77 for the amplitude in lead II and 0.67 for the dominant rate in lead V_1_ (Fig. 1A). When the cut-off values of the amplitude in lead II and dominant rate in lead V_1_ were 34.6 uV and 390/min, respectively, the sensitivity and specificity of a low amplitude in lead II and high dominant rate in lead V_1_ were 81.7% and 53.7%, respectively (Fig. 1B). Furthermore, the combined parameters of a low amplitude in lead II (RMS < 34.6 uV) and high dominant rate in lead V_1_ (DF ≥ 390/min) had a higher odds ratio in predicting LPeAF (OR 6.269, 95% CI 2.958–13.285, p < 0.001) than the stand-alone parameters of a low amplitude in lead II (RMS < 34.6 uV) or high dominant rate in lead V_1_ (DF ≥ 390/min) (Table 4).

**Table 4:** Multivariate analysis of the cut-off value depending on the characteristics of the f waves.

	Multivariate analysis (Model V)			Multivariate analysis (Model VI)		
	OR	95% CI	*p*	OR	95% CI	*p*
Age	1.03	0.994–1.068	0.103	1.036	1.002–1.071	0.037
Male	0.753	0.314–1.807	0.526	0.889	0.392–2.011	0.777
AF duration	1.285	1.106–1.493	0.001	1.241	1.080–1.427	0.002
Low amplitude (lead II) (<34.6 uV)	**5.642**	**2.432–13.087**	**<0.001**			
High dominant rate (lead V1) (≧390/min)	**3.456**	**1.562–7.648**	**0.002**			
Low amplitude in II and High dominant rate in V_1_				**6.269**	**2.958–13.285**	**<0.001**

The multivariate analysis in model V was adjusted for the age, gender, AF duration, low amplitude in lead II (<34.6 uV), and high dominant rate (DF) in lead V_1_ (≧390/min). The multivariate analysis in model VI was adjusted for the age, gender, AF duration, and combined parameters of a low amplitude size in lead II (<34.6 uV) and high dominant rate (DF) in lead V_1_ (≧390/min).

root mean square; RMS, dominant rate; DF.

## Discussion

In the current study, we found that the f-waves in LPeAF were short, fine and irregular, as compared to the other AF types. The distinct features of the f-waves in LPeAF were remarkable in leads II and V_1_: a significantly low amplitude in lead II and fast dominant rate and more irregularity in V_1_. Lead aVL, which is anatomically in proximity to the LA, was less powerful in discriminating LPeAF from other types, compared to II and V_1_. Only remarkable difference between PAF and PeAF was dominant rate in lead II, not in V_1_ and aVL. As LPeAF has a poor outcome with rhythm control by catheter or surgical ablation, it is clinically important to discriminate LPeAF from non-LPeAF to determine the treatment strategy. An analysis of the f wave characteristics in the 12 lead ECG could help differentiate the clinical types of AF.

### The detection of f-waves and their pathophysiologic implication

Atrial remodeling and fibrosis due to AF cause a decrease in the conduction velocity, prolongation of the PR interval, and decrease in the endocardial voltage^17^. This remodeling process continues to increase the heterogeneity of the atrial substrate and aggravates the dispersion of the atrial refractoriness. Atrial f-waves represent these complex changes and are traditionally classified as coarse or fine waves by their amplitude^18^, and fine waves are generally considered to be more disorganized, and to have an irregular pattern with a short atrial dominant cycle length. However, this classification has some limitations considering the various changes in the f-waves, because the atrial cycle length changes considerably due to the autonomic tone, heterogeneity of the atrial substrate, and diurnal variations. Even though that occurs, the properties of the f-waves acquired from a QRS cancellation can represent the electrical and mechanical characteristics of both atria^19–22^. In another assessment of the f-waves, a short dominant atrial cycle length was associated with a short atrial refractoriness, and an atrial substrate with those properties played a pro-arrhythmic role^19,23–25^. Previous studies have analyzed the f-waves in AF with a validation of the results through invasive measures. Those studies revealed that lead V_1_ is positioned at the nearest site to the right atrial free wall and well represents its waves. Additionally, the electrical waves measured in the coronary sinus, right atrial appendage, and esophagus have similar properties as lead V_1_^19,20^_._

### Clinical implication of the f-wave analysis

In the current study, the fibrillatory activity was three-dimensionally measured in the anteroposterior, superoinferior, and transverse axis through leads II, aVL, and V_1_, demonstrating somewhat different features among the clinical AF types according to the location and axis of each lead. It could be due to the different stages of the pathological changes and subsequent fibrillatory activity in the RA and LA. LPeAF has a more enlarged LA than PAF or PeAF in most cases, possibly resulting in low amplitudes in aVL and II. The finding that there is no significant difference in the irregularity index and dominant rate in leads II and aVL might suggest that the irregularity of the fibrillatory activity in the LA was similar in PAF, PeAF, and LPeAF. However, in the RA, PAF and PeAF might exhibit more flutter-like activity^2^, while LPeAF might have disorganized multiple electrical activities, as suggested by more irregular and fast dominant rates in V_1_^3^. A recent expert consensus statement recommended a classification system of PAF, PeAF, and LPeAF that can be used for future studies of catheter and surgical ablation of AF. LPeAF is defined by long-lasting events of for more than 1 year that are less responsive to a rhythm control strategy, including catheter and surgical ablation^14^. The prediction and identification of LPeAF at the time of the initial diagnosis has important clinical implications in preventing unnecessary treatments that would yield less in this clinical scenario. Our results show that patients with low amplitude f-waves in lead II and fine f-waves in lead V_1_ are likely to have long-lasting. In the current study, there were significant differences in age among PAF, PeAF, and LPeAF. It could be explained by the previous studies^26^ that the spectrum of AF types was related to aging process. Moreover, the identification of LPeAF could require a longer period time. Nevertheless, the multivariate analysis showed that difference of f waves characteristics are strongly associated with AF types.

### Methodological implications

The surface ECG is a non-invasive and simple diagnostic tool and has advantages for detecting serial changes. The extraction by a QRS cancellation, Fast Fourier Transformation, and power spectrum analysis were validated through an invasive electrophysiology study^20^, and after that, an analysis and quantification of the f-waves was performed with simple software. Furthermore, f-waves detected from the surface ECG may represent less localized atrial signals compared to an invasive electrophysiology study using bipolar electrodes that detect electrical activity within a very small distance, and the 12-leads can reflect signals obtained from various axes and directions of the atrium. Due to these advantages, previous studies have compared the characteristics of f-waves and the clinical outcome of rhythm control after cardioversion findings, and recent reports have noted that the AF rate (AFR)^27^, AF signal entropy (AFSE)^28,29^, and harmonic decay^27^ are independent factors of AF recurrences within 4 weeks after cardioversion.

### Limitations

Several limitations need to be acknowledged. The first, is that the f-wave variably changes according to the physical activity, autonomic tone, and diurnal variations. Furthermore, coarse f-waves can change to fine waves spontaneously due to these factors, and therefore it is important to repeatedly and serially measure the ECG. The second, we used only one mathematical formula to measure the irregularity, but there are several measures to express the severity of irregularity. It might have led to a less precise measurement of the irregularity. Nevertheless, the approximate entropy in the current study was enough to discriminate LPeAF from non-LPeAF in leads II and V_1_. Future studies regarding the relation of the f-wave analysis and clinical outcome of a rhythm control strategy will help clarify the significance of our findings. The third, we analyzed fibrillatory characteristics in only three leads, II, aVL, and V1 due to technical limitation. For more insightful information, further analysis in all leads should be performed in future. Finally, the exclusion of the patients whose data could not be acquired from the f-waves due to technical errors may have introduced a selection bias.

## Conclusions

A low amplitude and high dominant rate of the f-waves on the surface ECG could discriminate LPeAF from other types of AF.

## Methods

### Patient selection and definition of the AF type

The study protocol was approved by the Institutional Review Board of Yonsei University Health System in accordance with the tenets of the Declaration of Helsinki. We obtained informed consent from all enrolled patients. Consecutive patients newly diagnosed with AF by a surface 12-lead ECG. The AF type of all patients was classified by the American College of Cardiology (ACC), American Heart Association (AHA), and European Society of Cardiology (ESC) guidelines^15,16^. PAF was defined as sinus conversion of an AF rhythm of less than 7 days, and PeAF as that lasting for more than 7 days, which is unlikely to stop on its own^30^. In these cases, cardioversion or AADs could be used for the conversion of the AF to sinus rhythm, and if not successful or there were long-lasting events that continued for more than 1 year, the AF type was defined as LPeAF. So the prediction and identification of LPeAF take more time than PAF or PeAF. The age was significantly different among the three groups, patients with LPeAF were the oldest. To adjust the effect of age, we performed multivariate analysis including age and AF duration. ECGs obtained within initial 4 moths at the time of diagnosis of AF were analyzed in this study. All patients visited outpatient clinic regularly at 1, 3, 6, 9 and 12 months and then every 3 month thereafter or whenever symptoms occurred after the AF diagnosis. All patients underwent 12-lead ECG during every visit and 24- or 48-hour Holter recording and/or event recording at 3, 6, and every 6 months, according to the 2012 HRS/EHRA/ECAS Expert Consensus Statement guidelines. The AF duration was defined as the time since AF symptoms such as palpitation or chest discomfort started.

The exclusion criteria were as follows: (1) having no ECG data stored digitally in the electronic database system, (2) noisy A waves with baseline wandering in the 12 lead ECG, (3) taking AADs at the time of ECG, (4) V pacing rhythm in the ECG, (5) a medical history of valvular disease and past history of valve surgery or coronary bypass surgery, and (6) known structural heart diseases such as congenital heart diseases and valvular heart diseases with more than mild severity. We defined structural heart diseases as congenital heart diseases^31^, valvular heart disease more than moderate severity and the history of cardiac surgery. Finally, 224 AF patients were selected for f-waves analysis.

### Analysis of the chaotic activity of the fibrillary waves

#### Data acquisition

Standard 12 lead ECG data (GE Healthcare, Marquette, MAC5500, Waukesha, WI) digitally stored in the hospital ECG database were extracted. The paper speed was set to 25 mm/sec with a calibration of 10 mm/mV. The heart rate, PR interval, QRS, QTc, and P-axis were automatically measured by the ECG system. The Data was exported to.xml files, and converted into csv files through the Python program. Among the 12-lead ECG, leads II, aVL, and V_1_ were selected for the follow reason: The spatial position and axis of each lead in the 12-lead surface ECG could provide an estimation regarding the three-dimensional movement of the fibrillatory activity in the both atria (Sup. Fig. 1); Lead II having an axis aligned with the interatrial septum, reflects the inferior-superior axial movement of the electrical activation occurring in the both atria; The aVL axis which is perpendicular to lead II, could well reflect the electrical activity on the lateral side, that is, the left atrium (LA). V_1_ would be helpful to detect antero-posterior movement and in particular, due to the anatomical proximity to the right atrium (RA), allow for the detection of the electrical activity occurring in the RA.

#### Signal processing

**Figure 2:**
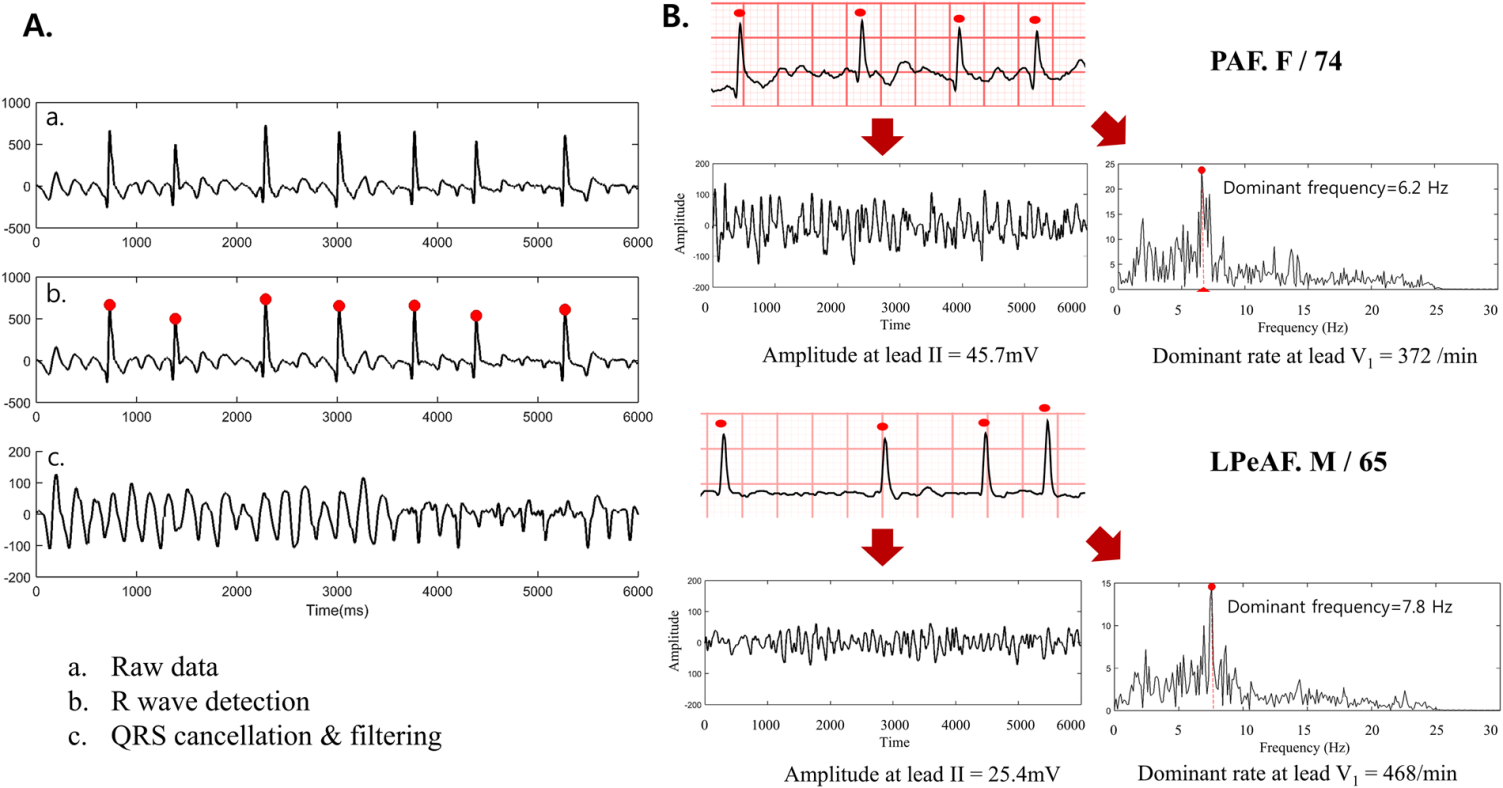
The extraction of the f-waves and a typical example. (**A)** Data acquisition and signal processing of the f-waves on the surface ECG. (**B**) Typical ECG of PAF with coarse (low dominant rate) f-waves and a high amplitude (high RMS), and of LPeAF with fine (high dominant rate) f-waves and a low amplitude (low RMS). PAF; paroxysmal atrial fibrillation, LPeAF; long-standing persistent atrial fibrillation, Dominant rate; DF, root mean square; RMS.

For off-line signal processing, a 10-s ECG recordings was preprocessed to reduce noise and interference^22,32^. The f-wave analyses were conducted in leads II, aVL, and V_1_ by a technician who was blinded to the clinical characteristics. A schematic presentation of the overall signal processing is depicted in Fig. 2. The raw signal is depicted in Fig. 2A-a. To avoid large residual errors, the signal sampling rate was increased from 500 Hz to 1000 Hz by a cubic spline interpolation method. To reduce the baseline wandering and high-frequency noise, the interpolated signal was filter by a Zerophase bandpass filter with a cut-off frequency of 0.5 and 30 Hz^33^. Then, a Pan Tompkins algorithm was applied for an automatic QRST detection (Fig. 2A-b), while the QRST complexes were removed using an adaptive singular value cancelation method (Fig. 2A-c)^34,35^. The final atrial f-waves were analyzed for the three parameters detailed below; (1) the root mean square (RMS; uV)^36^ as a parameter of the signal amplitude in the time-domain, (2) approximate entropy (ApEn)^37^ as a parameter of the signal irregularity in the time-domain, (3) and dominant frequency (DF; dominant rate/min)^38^ as a parameter of the power spectral density having the highest peak in the frequency domain. These 3 parameters were calculated for each studied lead (II, aVL, and V_1_). Finally, the signal was filtered by a Zerophase bandpass with a cut-off frequency of 3 and 30 Hz to suppress the residual QRST feature. The dominant frequency was calculated focusing exclusively within 3~9 Hz, since the power of the f-waves was mostly concentrated in the 4–9 Hz band of the power spectrum. We applied Piecewise linear correction of ECG baseline wander upon ECG signal^39^ to calculate an amplitude of baseline wander, not to subtract the wander. After normalization for amplitude of baseline wander of each patients, we excluded patients with upper 95 percentile, which was assumed as a threshold to classify ECG with noisy baseline wander from normal ECG. All signal processing was performed with a MATLAB (Mathworks Inc., Natick, MA, USA).

#### Parameters analyzing the features of the fibrillatory waves

The amplitude (RMS; uV) index was defined as the square root of the mean square, the arithmetic mean of the squares of the signal amplitude in the time-domain. The ApEn was calculated to quantify the irregularity of the fibrillary waves in the time-domain. For the ApEn, the sample size was set as 3, while the threshold was 3.5 times the standard deviation of the signal that was processed by QRS cancellation and appropriately filtered. The DF (dominant rate/min) was defined as the frequency occupying the highest peak in the power spectral density in the frequency-domain. The DF was mostly concentrated within the 4–9 Hz band of the power spectrum, and described the dominant rate per minute (dominate rate/min). Figure 2B shows a typical ECG of PAF with coarse (low dominant rate) f-waves and a high amplitude (high RMS), and of LPeAF with fine (high dominant rate) f-waves with a low amplitude (low RMS).

#### Statistical analysis

The non-normally distributed continuous variables were expressed by the median ± standard deviation of the parameters of the atrial waves. The statistical significance of the comparisons was assessed using a Mann-Whitney U test. To compare the clinical variables and atrial wave parameters (amplitude, ApEn, and dominant rate) between PAF, PeAF, and LPeAF, we used the Kruskal-Wallis test. Uni- and multi-variate logistic regression analyses were used for predicting LPeAF, with an adjustment for the age, gender, and AF duration (from the symptom onset to surface ECG acquisition date). Models I, II, and III represented the results of the multivariate analysis depending on each lead (II, aVL and V_1_) after an adjustment. The cut off values for the amplitude in lead II and dominant rate in lead V_1_ (model V, VI), which best differentiated LPeAF from non- LPeAF, were determined by an algorithm for the maximization of the hazard ratio. A p-value < 0.05 was considered statistically significant.

## Supplementary information

Supplementary table and figure
