# Supplementary material for: Early differentiation of long-standing persistent atrial fibrillation using the characteristics of fibrillatory waves in surface ECG multi-leads
Source: Sci Rep. 2019 Feb 26;9:2746. doi: 10.1038/s41598-019-38928-6 (PMC6391406; doi:10.1038/s41598-019-38928-6)

Early differentiation of long-standing persistent atrial fibrillation using the characteristics of fibrillatory waves in surface ECG multi-leads

Junbeom Park, MD., PhD.,[1] Chungkeun Lee, PhD.,[2] Eran Leshem, MD., MHA.,[3] Ira Blau,[4] Sungsoo Kim, MD., MS.,[5] Jung Myung Lee, MD.,[6] Jung-A Hwang,[7] Byung-il Choi, MD.,[4 7] Moon-Hyoung Lee, MD., PhD.,[7] Hye Jin Hwang MD., PhD. [3,7]

[1]Department of Cardiology, College of Medicine, Ewha Womans University

[2] Cardiovascular Devices Division, National Institute of Food and drug safety Evaluation, Cheongju-si, South Korea

[3]Cardiovascular Division, Department of Medicine, Beth Israel Deaconess Medical Center, Harvard Medical School, MA, USA

[4] Department of Cardiology, Medical College of Wisconsin, Milwaukee, WI, USA

[5] College of Medicine, Yonsei University, Seoul, South Korea

[6] Department of Medicine, Graduated School, Kyung Hee University, Seoul, South Korea

[7] Department of Cardiology, Yonsei University Health System, Soul, South Korea

Supplementary Table 1. Difference in the fibrillary waves according to the lead (II, aVL, and V_1)

	Lead II	Lead aVL	Lead V_1	p	(1)	(2)	(3)
**Number of patients**	208	192	193				
**Amplitude (RMS, uV)**	37.50±17.46	23.07±8.40	44.79±17.03	<0.001	<0.001	<0.001	<0.001
**Irregularity (ApEn)**	0.12±0.02	0.13±0.02	0.12±0.02	<0.001	<0.001	0.007	0.84
**Dominant rate (DF, rate/min)**	316.10±124.43	340.27±107.81	353.06±117.72	0.006	0.157	0.234	0.001

p, p-value by the Kruskal-Wallis test; **(1)**, p-value in II vs. aVL; **(2),** p-value in aVL vs. V1;**(3),** p-value in V_1 vs. II

Supplementary figure 1. The relationship between the surface ECG and cardiac anatomy

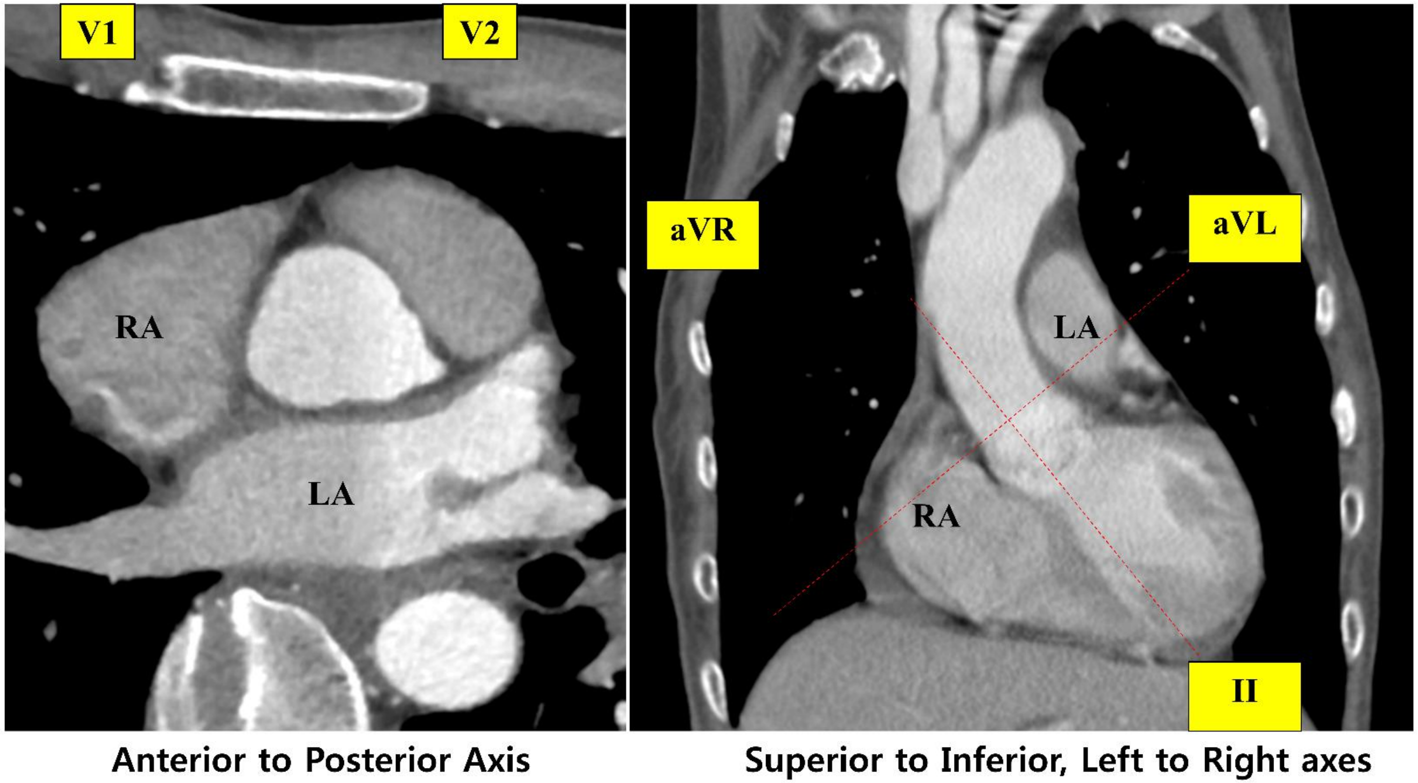

Supplement: Supplementary file 1 — Supplementary table and figure [file 41598_2019_38928_MOESM1_ESM.pdf]
